# Toward Learning Trustworthily from Data Combining Privacy, Fairness, and Explainability: An Application to Face Recognition

**DOI:** 10.3390/e23081047

**Published:** 2021-08-14

**Authors:** Danilo Franco, Luca Oneto, Nicolò Navarin, Davide Anguita

**Affiliations:** 1 Department of Computer Science, Bioengineering, Robotics and Systems Engineering, University of Genoa, Via Opera Pia 11a, 16145 Genova, Italy; danilo.franco@edu.unige.it (D.F.); davide.anguita@unige.it (D.A.); 2 Dipartimento di Matematica “Tullio Levi-Civita”, University of Padua, Via Trieste 63, 35121 Padova, Italy; nnavarin@math.unipd.it

**Keywords:** trustworthy artificial intelligence, deep neural networks, Algorithmic Fairness, learning fair representation, privacy-preserving machine learning, Homomorphic Encryption, explainable artificial intelligence, attention maps, dimensionality reduction

## Abstract

In many decision-making scenarios, ranging from recreational activities to healthcare and policing, the use of artificial intelligence coupled with the ability to learn from historical data is becoming ubiquitous. This widespread adoption of automated systems is accompanied by the increasing concerns regarding their ethical implications. Fundamental rights, such as the ones that require the preservation of privacy, do not discriminate based on sensible attributes (e.g., gender, ethnicity, political/sexual orientation), or require one to provide an explanation for a decision, are daily undermined by the use of increasingly complex and less understandable yet more accurate learning algorithms. For this purpose, in this work, we work toward the development of systems able to ensure trustworthiness by delivering privacy, fairness, and explainability by design. In particular, we show that it is possible to simultaneously learn from data while preserving the privacy of the individuals thanks to the use of Homomorphic Encryption, ensuring fairness by learning a fair representation from the data, and ensuring explainable decisions with local and global explanations without compromising the accuracy of the final models. We test our approach on a widespread but still controversial application, namely face recognition, using the recent FairFace dataset to prove the validity of our approach.

## 1. Introduction

Trustworthiness in artificial intelligence (AI) stands out as one of the main problems to be addressed in developing the future of the modern technological societies [1]. One of the first stances that depicted the necessity of deploying trustworthy information and communications technologies goes back to 1999 with “Trust in Cyberspace” [2], where researchers showed that governments started to become dependent on possibly unreliable algorithms for operating their critical infrastructures, such as communication, transportation, and power distribution [3]. Analogously to what has been done in the past for these infrastructures, there is nowadays a need to address trustworthiness in AI systems as a holistic property able to guarantee fundamental rights, encapsulate the ethical principles of the society, enforce resilience to disruption, and cope with human errors or hostile attacks [4]. The resulting benefits are numerous and multifaceted. For example, it can contribute to increasing well-being both on a collective and an individual level, for example by generating wealth [5] or taking care of tedious or dangerous tasks [6]. Moreover, it can promote fairer behaviors toward social and political equality [4].

In general, this ambitious objective cannot be reached in a single step, but there is a need to first face specific sub-problems and then combine the results toward a more holistic approach [7]. In the context of AI, a fundamental building block is the ability to learn from data by means of machine learning (ML)-based technologies [8]. This ability allows us to make predictions based on historical data supporting decision makers (human or autonomous) [9]. Models learned from data have been shown to deliver very accurate results in recent years, outperforming human abilities in some specific applications [10,11,12] with the use of increasingly complex ML algorithms on the increasing number of available data [13]. Simultaneously, researchers have begun to show the drawbacks of rushing towards these accuracy levels: models have started to also learn the human biases and misbehavior [14,15,16], to break the privacy of the single individuals [17,18], to show limited robustness to (malicious) data perturbations [19,20,21,22,23], and to be less and less understandable, undermining the fundamental right of explanation principle [24]. For these reasons, researchers have started to study these problems separately, developing the fields of Algorithmic Fairness [25,26,27], Privacy-Preserving Data Analysis [28], Adversarial Machine Learning [29], and Explainable Machine Learning [30,31,32], respectively. Unfortunately few works in the literature have tried to address more than one of these problems simultaneously. Some have tried to face two of them: for example some works combine fairness with privacy [33,34,35,36,37,38,39,40,41], others [42,43,44] combine adversarial learning with fairness, fairness with explainability [45,46,47], adversarial learning with explainability [48,49], and adversarial learning with privacy [50,51,52,53]. For this reason, in this work, we drive toward the development of systems able to ensure trustworthiness by delivering privacy, fairness, and explainability by design.

Privacy requires protecting the data of the single individuals along with all the information generated during the entire data lifecycle [4]. It is easy to explain the practical necessity of privacy guarantees, especially in those applications where digital records directly contain (or can be exploited to infer) highly sensitive information, such as gender, ethnicity, or sexual or political orientations [28,54]. For this reason, it is required to cope with the problem of developing ML models able to simultaneously extract useful and actionable information from data and not violate the privacy of single individuals. Algorithmic Fairness requires the outputs of ML-based models to not depend on sensitive attributes (e.g., gender, race, and political/sexual orientation) [26,55]. In fact, datasets may contain historical biases (e.g., discrimination against historically mistreated subgroup in the population) or may suffer from a coarse view of the modern societies (e.g., underrepresented subgroups). ML models trained on those biased data may exacerbate unfairness, generating a cascade effect [4,7,56]. For this reason, it is required to cope with the problem of developing methods to mitigate such biases. Explainability in ML is the ability to provide an explanation for the output of an ML-based model [30,32,57]. Explanations can be local (i.e., why the model gave a particular output provided a particular input) or global (i.e., what the model actually learned from data) [32,57]. An increasing level of explainability in the decision-making process also facilitates model traceability, which, in turn, could help reveal the possible points of failure and prevent future mistakes [4,57]. Making state-of-the-art ML-based models (i.e., deep neural networks) explainable is a quite challenging task, which needs to be directly addressed to cope with the right of explanation [24,58] but also for understanding other problems (i.e., unfair behavior, limited robustness, or leaks in privacy of the model itself [30,57]).

In this work we show how to adapt and combine state-of-the-art approaches with the purpose of learning from data under privacy, fairness, and explainability requirements. In particular, we show that it is possible to learn from data, leveraging on deep pretrained models [59,60], simultaneously preserving the privacy of the individuals thanks to the use of Homomorphic Encryption (HE) [61], ensuring fairness by learning a fair representation from the data [62,63,64], and delivering explainable decisions with local and global explanations [57] without compromising the accuracy of the final models. Then we will test our approach on a widespread and controversial problem, namely facial recognition, using the recent FairFace [65] dataset to prove the validity of our approach. In fact, deep pretrained networks allows one to easily and inexpensively extract a representation vector that can be then used and fine-tuned for a specific application at hand [59,60,66]. This avoids the need to design and train from scratch a new network, which would require a huge number of data and computational resources, which is seldomly available in practical applications [67]. Nevertheless, even if an architecture is already available (with its tuned weights), we need to find smart ways to fine tune the network with limited data and a number of increasing constraints [68], especially when new data become available (e.g., the phenomena is changing) or new requirements arise (e.g., privacy and/or fairness requirements). HE has gained a lot of attention in the field of privacy-preserving machine learning since it allows working on encrypted data seamlessly, as the computations are performed on their original non-encrypted version [69]. Three main approaches exist (i.e., Partially, Somewhat, and Fully HE [70]), for which there is a tradeoff between the number of recoverable computations and the operations type allowed. For our purpose, namely ML-related applications, Somewhat HE is the most exploited approach, since it delivers the best trade-off [71] for our application. HE, on one hand, allows ensuring the privacy of the single individual, especially in the commonly adopted case where the computing and memory resources are outsourced to a third-party service provider, but on the other hand dramatically increases the computational requirements and reduces the possible network architectural choices [72,73]. Algorithmic Fairness deals with the problem of ensuring that the learned ML does not discriminate subgroups in the population using pre- in- and post-processing methods [26,74]. When deep models are exploited, such as in our use-case, learning a fair representation from the data (instead of simply trying to make the models fair) has been shown to be the best approach [62,63,64,75]. Still, these approaches can hardly be combined with HE since not all the operation and architectural choices are allowed due to the intrinsic limitations of HE [72,76]. For this purpose, in this work, we show that a particularly simple yet effective constraint for learning fair representation [64,77] can be combined with deep models and HE to deliver deep, fair and private models. In our work, fairness is measured according to the Demographic Parity (DP) [78], which requires the probability of the possible model decisions to be independent of the sensitive information. Finally, to deliver both local and global explainability, we rely on two state-of-the-art approaches. For local explainability, we exploit the attention maps of Deep Neural Networks through the Grad-CAM [79] algorithm, which highlights the most significant input features for a particular prediction. For global explainability, we will rely on both average attention maps and a dimensionality reduction algorithm, namely t-SNE [80,81]. Since these methods are straightforwardly applicable in conjunction with HE, they can be used to check the effect of the fairness constraints on what the deep models actually learned from data. In this sense, we are using explainability as a provision for the user right of explanation and as an inspection mechanism for the model creator as well.

The rest of the paper is organized as follows. Section 2 summarizes the works in the literature related to our research. Section 3 reports some preliminary notions instrumental for understanding our work. Section 4 presents the proposed method. The results of applying the method proposed in Section 4 on the face recognition task by means of the FairFace dataset are presented in Section 5. Section 6 concludes the paper.

## 2. Related Works

This section is devoted to a brief review of the works related to the context of our paper. For what concerns the fairness mitigating methods, they are usually categorized depending on the way they actually work [25,26,74,82]. For classical (i.e., shallow) ML models [83] trained on manually engineered features based on domain knowledge, we have three main families of mitigation methods: pre-, in-, and post-processing. Pre-processing methods try to remove the biases in the data so that any learning algorithm trained on those cleaned data should generate a fair model. In-processing methods impose the fairness constraints directly into the learning phase, enforcing fairness in the model’s inner structures. Finally, post-processing tracks the output of an already trained model to make it more fair. When it comes to dealing with deep learning [66], where the ML models try to simultaneously extract a synthetic yet expressive representation of the raw data (e.g., images or natural language) without any prior knowledge or human intervention, it has been recently shown [62,64,84,85,86] that the best approach is to learn a so-called fair representation. This fair representation can be reused to train other models which will be, again, fair by-design.

For what concerns the methods for making ML models privacy-aware/compliant, they can be divided into anonymization techniques, perturbation techniques, and distributed protocols [28,87,88]. The anonymization techniques try to maintain the privacy of the data subjects by obscuring personally identifying information within a dataset while preserving data utility. k-Anonymization [89], l-Diversity [90], and t-Closeness [91] are the most known approaches for anonymization. Perturbation techniques exploit noise to corrupt the data, the ML algorithm, or the learned model quantifying the disclosed information in terms of the power of the noise. Differential Privacy [92] is the most prominent theoretical framework for the perturbation techniques. Anonymization and perturbation techniques assume the existence of a trusted curator of the data. When this is not available, we need to use distributed protocol techniques [93].

For example, Federated Learning [94], one of the prominent approaches in distributed protocol techniques, requires participants to train their models privately and then to share the results. However, privacy may still be compromised once the local parameters are shared, such as the updating gradients, which that may disclose information on the user’s private data. Recent works mix the use of Federated Learning with different HE schemes to address these issues [95,96]. In fact, HE recently attracted a lot of attention since it allows one to work on encrypted data as the computations are performed on their original non-encrypted version [97,98]. Consequently HE, contrarily to anonymization and perturbation techniques, entirely preserves utility and, contrarily to other simple distributed protocols techniques, automatically guarantees preserving the privacy of the single individuals. The major drawback of HE is its high computational overhead and the limitations for some operations [72,73]. In particular, three possible approaches are defined: Partially, Somewhat, and Fully HE. Partially HE benefits from an unlimited number of computations, but only one operation is allowed (multiplication: RSA [99], addition: Pailler [100]). Somewhat HE allows for multiple operations but suffers from a limited number of computations due to an increasing amount of computations-derived noise (BFV [101], CKKS [102]). Fully HE allows both a multiple number of operations and an unlimited number of computations, but generally suffers from huge computational costs (Gentry’s [103]).

Concerning explainability, as a general rule, the complexity of a ML model is inversely proportional to its level of clarity and interpretability [31,57,104]. One solution to this issue is to design and implement intrinsically explainable algorithms. Alternatively, another widely used possibility is to build a highly accurate black-box model and then design a post-hoc explanation. Post-hoc explanation methods can be categorized into two families: global and local explanations. The former aims at understanding the entire logic of a system and retracing back the predictions’ reasoning, while the latter is specific to a single instance and tries to justify single decisions. Post-hoc explanations are often model-agnostic, meaning that they are not tied to a particular type of ML systems [31,57]. Since explanations are mostly meant to be exploited by humans, they are usually meant to be visualized. For example, LIME [105] exploits a local surrogate model to explain the reason for a particular output. Relative to the context of computer vision, attention maps (e.g., using the Grad-CAM [79] algorithms) allow one to identify the influence regions of an image that most contribute to a particular decision. Both approaches can be used either as local methods of explanation if applied to only one image or as global methods of explanation if applied to a subset of the data [57]. In addition, dimensionality reduction methods, such as t-SNE [80], can be exploited as usually global explanations since they allow understanding how data, representation, and decisions are distributed and how changes in models or in the constraints (e.g., fairness) influence this distribution.

Many works [33,34,35,36,37,38,39,40,41] have tried to address fairness and privacy guarantees together. Kilbertus et al. [34] is one of the first proposals that addressed the need for combining fairness requirements with privacy guarantees. Their approach is to mitigate Disparate Impact [106] (i.e., discrimination due to the correlation between sensitive and non-sensitive attributes) without disclosing sensitive information through secure multi-party computation. Jagielski et al. [35] builds on top of the previous work, stating that secure multi-party computation offers insufficient privacy guarantees due to the possible leakage of sensitive attributes. They provide a different approach based on Differential Privacy, where privacy is guaranteed through an injectable amount of noise able to mask the presence of a protected individual in a particular dataset. In this direction, other works [36,37,38,39] aimed to learn fair and differentially private ML models. Cummings et al. [36], while showing that it is impossible to achieve both differential privacy and exact fairness without non-trivial accuracy, provides a Probably Approximately Correct [107] learner that is differentially private and approximately (with high probability) fair. Xu et al. [38] presents two methods for achieving Differential Privacy and Algorithmic Fairness within a logistic regression framework through Functional Mechanism [108] that achieves privacy and fairness by injecting Laplacian noise into the model objective function. Mozannar et al. [37] proposes a two step algorithm where the first phase finds an approximately non-discriminatory predictor, while the second produces a final predictor with Local Differential Privacy guarantees [109]. Besides all the characteristics of standard Differential Privacy, Local Differential Privacy excludes the possibility in which an adversary is able to learn any sensitive information about a particular data point. On a related note, Bagdasaryan et al. [39] observed that standard Differential Privacy methodologies, such as gradient clipping and noise addition [110], yield disparate impact for underrepresented subgroups: the accuracy for those classes in a privacy-enhanced model tends to deteriorate more when compared to the non-private case. In this sense, this work empirically demonstrated that carelessly managing Differential Privacy will end up in exacerbating unfairness, hence supporting the need for alternative choices for pursuing privacy-preserving ML. Oneto et al. [33], instead, studies the privacy and fairness properties of randomized algorithms, proving that in this framework, it is possible to naturally impose fairness (measured with a generalized notion of fairness contemplating Equal Opportunity, Equal Odds, and Demographic Parity) and quantify the amount of disclosed information (via differential privacy) with theoretical guarantees. Unfortunately, the approach is still quite theoretical and practical evidence is still missing.

Some other works have tried to discuss the need for theoretical and practical ethical ML-enforcing privacy, fairness, and explainability properties [4,111,112,113]. Nevertheless, to the best of the authors’ knowledge, in the literature, there are no works that simultaneously focus on enforcing privacy (especially HE), fairness, and (local and global) explainability in a theoretically grounded way and with actual empirical evidence in a realistic application.

In our work, we focus on a common face recognition problem using the recently released FairFace dataset [65]. Facial recognition is becoming a widespread and controversial tool used in many different contexts (e.g., from recreational activities to policing). Its popularity has increased so rapidly over the last few years that facial recognition software is commonly also used by government agencies [114]. Nevertheless, much recent evidence [115,116,117] shows how these algorithms can be biased against black people and women. In reaction to these issues, according to CNN [118], some governments banned the usage of facial recognition systems in law enforcement agencies and public-facing businesses. Making face recognition algorithms more trustworthy (fair, private, and explainable) would greatly improve the public opinion of them and their general acceptance.

Historically, traditional methods for facial recognition attempted to extract handcrafted shallow features (e.g., Viola-Jones [119], Gabor [120], LBP [121]), and, before the advent of deep ML models, they represented the state of the art for classical benchmark datasets [122]. Deep learning models have recently been shown to outperform these classical methods, being more robust to changes in illumination, face pose, aging, expressions, and occlusions [123]. In particular, Convolutional Neural Networks (CNN) are designed to be particularly proficient in facial recognition tasks and image recognition in general [124,125], employing a series of convolutional, pooling, and activation layers for extracting expressive representation from the input images. Moreover, the possibility of exploiting pretrained networks (e.g., LeNet [126], AlexNet [127], GoogleNet [128], VGGNet [129], and ResNet [130]) as-is or fine-tuned represents the state-of-the-art approach for different computer vision tasks [124]. In this work, we rely on the VGGNet architecture since it offers a good trade-off between accuracy, computational resources, and ease of use. Moreover, VGGNet differs by a few percentage points in accuracy from other state-of-the-art deep neural networks [122,131,132,133].

## 3. Preliminaries

Let us consider the probability distribution μ on I×S×Y, where I is the input space, S={1,2} identifies a binary sensitive variable (in our case the binary gender, i.e., male and female) and Y={0,1} is a binary label (in our case < and ≥ of 30 years old). For S, our method easily extends to multiple sensitive variables and continuous variables, but to ease the presentation, we consider only the binary case in the paper. In our work, $I\subseteq R^{h\times w\times 3}$ is the space of all RGB images of human faces, where *h* and *w* are the height and width of the image, while the third dimension defines the three standard color channels (Red, Green, and Blue). Let $D={\left(I_{i},s_{i},y_{i}\right)}_{i=1}^{n}\in {\left(I\times S\times Y\right)}^{n}$ be a set of *n* samples from μ. For each s∈{1,2}, let $D_{1}=\left\{\left(I,s,y\right)\in D\;|\;s=1\right\}$ and $D_{2}=\left\{\left(I,s,y\right)\in D\;|\;s=2\right\}$ be the set of samples in the first and second group, respectively. The goal is to learn a model h:Z→Y able to approximate P{y|Z} where Z∈Z may contain (Z=I×S) or not (Z=I) the sensitive attribute, depending on the specific regulation [134,135]. The ability of h of approximating P{y|Z} is measured with different indices of performance P(h) based on the required properties and the different tasks under examination [66]. For example, in binary classification P(h) can be the Accuracy or the Mean Square Error.

Within the context of the increasingly popular deep ML models, h can be described as a composition of simpler models m(r(Z)), where $m:R^{d}\to Y$ is a (non-)linear function and $r\left(Z\right)\in R^{d}$ is a function mapping the input data into a vector, usually referred to as the representation vector. Note that r can be a composition of functions as well $r:r_{l}\circ \cdots \circ r_{2}\circ r_{1}$, for example, in a deep neural network of *l* layers [66]. In other words, the function r creates a compact and expressive description of the input space that can deliver high accuracy when used by m to solve a specific task. r, learned in a particular context, can be reused by many models m as it is or fine tuned for the specific task at hand.

According to Algorithmic Fairness, we expect the model h to be fair with respect to one or more notions of fairness [26]. As recently theoretically studied in [64] and empirically demonstrated in many works [62,63,136,137,138,139], when deep learning models are developed, learning a fair representation actually allows one to make the entire network fairness-aware. Intuitively, this fair representation could be subsequently exploited by other ML models, for example, within the context of Transfer Learning [140], enforcing fairness by-design. In our work, we require the representation vector to satisfy the DP constraint [78]. Other notions of fairness could be exploited in this paper such Equal Opportunity and Equal Odds [141], but this extension is straightforward and out of the scope of this paper.

(1) $$\begin{array}{c} P_{Z}\left\{r\left(Z\right)\in C\;|\;s=1\right\}=P_{Z}\left\{r\left(Z\right)\in C\;|\;s=2\right\},\;\forall C\subseteq R^{d}, \end{array}$$

namely, the two conditional distributions of the representation vector should be equal with respect to the sensitive attribute. The constraint of Equation (1) directly implies that any model m learned on top of a fair representation will be again fair

(2) $$\begin{array}{c} P_{Z}\left\{m\left(r\left(Z\right)\right)=1\;|\;s=1\right\}=P_{Z}\left\{m\left(r\left(Z\right)\right)=1\;|\;s=2\right\}. \end{array}$$

The performance P(h) of the final models h will be evaluated with the accuracy metric (${\mathrm{ACC}}_{y}\left(h\right)$), namely percentage of correctly classified samples, computed on the test set (i.e., data not exploited to train h) [142]. Exploiting Equation (1), the fairness of the final models h will be measured by means the Difference of Demographic Parity (DDP) [64]

(3) $$\begin{array}{c} \left|\frac{1}{\left|D_{1}\right|}\sum_{\left(Z,y\right)\in D_{1}}\left[h\left(Z\right)=1\right]-\frac{1}{\left|D_{2}\right|}\sum_{\left(Z,y\right)\in D_{2}}\left[h\left(Z\right)=1\right]\right|, \end{array}$$

where the Iverson bracket notation is used.

We will rely on HE for enforcing privacy guarantees. In linear algebra, a homomorphism is a transformation between two algebraic structures that preserves the defined operations. For example, let ϕ:A→B be a homomorphic map between two sets A and B with the same algebraic structure, if ⊕ is a binary operation on that structure, then $\phi \left(A_{1}⊕A_{2}\right)=\phi \left(A_{1}\right)⊕\phi \left(A_{2}\right)$, $\forall A_{1},A_{2}\in A$. Hence, HE is an encryption protocol that relies on homomorphic transformations obtained through the definition of public (i.e., encryption) and private (i.e., decryption) keys. Thanks to the property of homomorphism, some operations can be performed on the encrypted data as they were carried on the non-transformed version preserving the privacy of the original data. Specifically, we will rely on Somewhat HE using the CKKS scheme, which allows a bounded number of computations limited to addition, multiplications, and rotations. The CKKS algorithm defines four phases: encoding, encrypting, decrypting, and decoding [102]. First, the input data, which consist of a vector of real values, are encoded into a polynomial (i.e., the plaintext) of degree *p*, where *p* is a power of 2. CKKS works with cyclotomic polynomials from the Ring theory because they offer a good trade-off between security and efficiency [102]. The plaintext is then encrypted into a pair of different polynomials (i.e., the ciphertext) through the use of a public encryption key. The homomorphism of this encryption is achieved thanks to the theory of Ring Learning With Error [143], where, of particular interest for this work, addition and multiplication are preserved. While additions cause no obstacles, multiplications increase the noise kept in the pair of ciphertexts; therefore, only a limited number of products is allowed. However, higher polynomial degrees allow for wider computational bounds, yet they are more expensive in terms of processing and memory requirements. Once the required computations are performed, the pair of ciphertexts can be reverted back first to the plaintext polynomial through the use of the secret decryption key, and then to the vectors of values through the final decoding phase. The output vectors will yield approximate results, close to the real solution thanks to the property of homomorphism. The polynomial degree *p* must be chosen as small as possible to guarantee the correctness of the results without increasing too much the computational requirements [102].

In order to improve the readability of the technical parts, we added the list of notations in Table 1 that are exploited in the paper.

## 4. Proposed Method

In this section we will present our approach to learn private, fair, and explainable deep ML models. In particular, we will start presenting our approach to private deep ML models based on HE showing the limitations that are implied in terms of computations and operations (Section 4.1). Then we will present the chosen architecture, with particular reference to the exploited facial recognition application. The proposed architecture slightly differs from the classical one due to the handling of the HE limitations (Section 4.2). Following this analysis, we will show how to impose the fairness constraint, again taking into account the limitation imposed by HE, using the fair representation framework (Section 4.3). Finally, we will empower the proposal with explainability properties that will be used also to understand what is learned from the deep model and whether the fairness constraint actually changes how and what the architecture perceives from the images (Section 4.4).

### 4.1. Making the Model Private

As previously mentioned, to enforce privacy, we relied on HE during both the training and forward phases of the deep ML models. During training time, each sample is encrypted following the CKKS [102] scheme to a high order polynomial that masks the real data attributes and labels. Then these encrypted values are fed to the DNNs, which output encrypted predictions. Thanks to the homomorphism property, the masked labels and predictions can be compared through a loss function. The loss function needs to be expressed in terms of additions and multiplications (the only operations allowed by CKKS) so a polynomial loss function is the most natural choice (e.g., the Mean Square Error [144]). During the training phase, we rely on Gradient Descent algorithms [66,145], which natively require us to compute just additions and multiplications. This is true only if the architecture of the deep ML model does not contain special (non-polynomial activations) functions whose derivative cannot be expressed easily with additions and multiplications (this limits our architectural choices; e.g., the widely-used RELU activation function cannot be deployed). Belonging to Somewhat HE, CKKS adds a certain amount of noise to the encrypted data, which increases with the number of stacked layers [72,76]. This fact also limits the depth of the network. Finally, the CKKS scheme heavily increases the memory and computational requirements for storing and processing the data, further limiting the architectural choices and the number of data that we can use to train the network. Note that the privacy of the deep network can be enhanced by also encrypting the weights of the network [146] (e.g., to avoid or at least mitigate adversarial attacks (https://blog.f-secure.com/mitigations-against-adversarial-attacks (accessed on 11 August 2021)). The process of encryption/decryption is performed through the python TenSEAL [147] library for the CKKS scheme which easily allows the integration with common deep ML software frameworks like PyTorch [148]. Other libraries, even more efficient, exist [149,150], but they can be hardly combined with deep ML software frameworks.

### 4.2. The Architecture of the Deep Model

In this work, we will exploit the VGGNet-16 [129] (Configuration D) as the architecture for face recognition. VGG-based networks exploit deep architectures, leading to quite accurate results for a variety of different tasks while maintaining relatively low computational requirements thanks to the use of small filters. In fact, stacking convolution layers with small kernels seems to be preferable to using a single layer with larger kernels, providing computational and generalization advantages [129]. Moreover, the use of multiple stacked layers allows tone o easily increase the nonlinearity harnessed by the network by adding an activation function at each intermediate step. The VGGNet-16 embeds the input data in a 25,088-dimensional vector space by means of 14 million parameters, which allow lynear state-of-the-art results to be achieved in multiple facial-recognition-related tasks [122,131,132,133]. Because of such complexity, the VGGNet-16 deployed in this work has been pretrained on the face recognition dataset VGG-Face [151].

However, in this work, we will need to depart from the standard end-to-end use of deep learning models due to the limitation imposed by HE as expressed in Section 4.1. The convolutional layers need to be kept fixed, i.e., used just to extract the embedding. In fact, fine-tuning them would require using end-to-end HE for the whole architecture, resulting in an intractable problem from the computational point of view. Actually, the 25,088-dimensional embedding cannot be directly used, for the same reason above, but we have to shrink it in a much smaller embedding, i.e., a 32-dimensional, by means of a dense layer with Sigmoidal activation. The parameters of this layer, along with those of the last convolutional layer, are initially pretrained on the FairFace dataset. To give an idea of why we chose 32 as the dimensions of the embeddings, we point out that managing 1000 images embedded in this 32-dimensional vector requires approximately 30 GB of memory (see details in Section 5).

The actual learning phase was conducted starting from these fixed 32-dimensional embedding using a single hidden layer architecture empowered with HE. The 32-dimensional vector is fed into a 16-dimensional dense layer with a square activation function, which complies with the CKKS limitation. The output of this layer is fed into a 2-dimensional dense network with linear activation. We did not use a single output neuron for being able to exploit the Grad-CAM visualization algorithm (see Section 4.4). The parameters of these last layers are randomly initialized according to a Gaussian distribution N(0,0.01).

**Figure 1:**
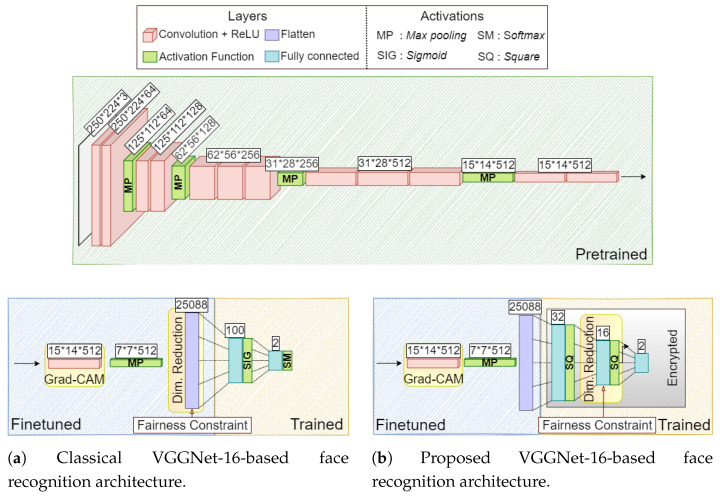
Classical and proposed architectures for facial recognition. Figure shows the pretrained, the trained, and the fine tuned layers, where the HE is employed, the fairness constraints, and the visualization methods are applied.

Figure 1 represents both a classical architecture [45] (Figure 1a) and the proposed one (Figure 1b) for facial recognition.

### 4.3. Making the Model Fair

As reported previously, many different approaches exist to impose the fairness constraint of Equation (1). In particular, following the fair representation principle [45], we propose the formulation of a Tikhonov regularizer F(h) for balancing the possibly biased performance index P(h) (Section 3) in the cost function as follows:

(4) $$\begin{array}{c} h^{*}=\arg\min_{h}P\left(h\right)+\lambda F\left(h\right), \end{array}$$

where λ∈[0,+∞) trades off accuracy and fairness, as we will also see in Section 5. Note that the constraint could have been imposed using the Ivanov philosophy [152], and the results would be the following optimization problem

(5) $$\begin{array}{c} h^{*}=\arg\min_{h}\;P\left(h\right),\;s.t.\;F\left(h\right)\leq \eta , \end{array}$$

where η∈[0,1] regulates the level of accepted fairness, which is cognitively more close to the problem of imposing a certain level of fairness to the final model. Nevertheless, note that, for some values of η and λ, the two problems of Equations (4) and (5) are equivalent, but Problem (4) is much less computationally demanding with respect to Problem (5) [153]. Note also that setting η=0 in Problem (5) (or λ→+∞ in Problem (4)) to impose the DP does not guarantees fairness in terms of generalization since Problem (5) (or Problem (4)) exploits empirical quantities. Setting η∈[0,1],λ∈[0,+∞) allows one to avoid overfitting the particular sample.

The concept of learning a fair representation is expressed, in this context, by imposing the regularizers on the last layer of the representation, namely F(h)→F(r). This translates, in the classical architecture, into imposing the constraint in the last convolutional layer as the most effective strategy [45] (see Figure 1a), while, for the proposed architecture, in imposing the constraint in the last tunable representation layer, i.e., the 16-dimensional last hidden layer (see Figure 1b).

Unfortunately, the fairness constraint of Equation (3) is practically hard to handle, and it is necessary to approximate it by defining effective yet computationally efficient alternative regularizers, which also have to meet the HE limitations. In the literature, different approaches have been proposed, and the most effective ones appear to be the one reported in [64]. The authors of [64] propose three different regularizers: one based on convex approximation and relaxation of the constraint of Equation (1), one based on the squared Maximum Mean Discrepancy [154], and one based on the Sinkhorn divergence [155].

Because of the limitations imposed by HE, the approaches based Maximum Mean Discrepancy and Sinkhorn divergence cannot be effectively employed. We rely on the convex approximation and relaxation of the constraint of Equation (1) proposed by [64] where the regularizer assumes the following form:

(6) $$\begin{array}{c} \mathrm{AVG}\left(r\right)={∥\frac{1}{\left|D^{1}\right|}\sum_{\left(Z,y\right)\in D^{1}}r\left(Z\right)-\frac{1}{\left|D^{2}\right|}\sum_{\left(Z,y\right)\in D^{2}}r\left(Z\right)∥}^{2}. \end{array}$$

Note that this convex approximation and relaxation is simply the first-order approximation of Equation (1). Note that if the chosen architecture can be handled with H, this regularizer added in the cost function simply adds a term which can be computed with sum and multiplications, such as its derivatives.

### 4.4. Making the Model Interpretable

In order to provide both local and global explanations and to visualize how the CNNs react to the input images, we analyze the attention regions obtained through Grad-CAM [79]. Specifically, Grad-CAM extracts attention maps (i.e., heatmap images) that highlight the most influential features for a particular supervised task. They can be used as a local explanation method if applied to a single instance or global if the result is averaged over a subset of the instances (e.g., over all the men older than 30 years old). When dealing with fairness, attention maps can underline any divergence in the representations between different protected groups.

By fixing a classification prediction target y∈Y (i.e., the output neuron of the network corresponding to particular class), a non-normalised network score $y_{n}$ for *y* (i.e., prior to the final Softmax activation for classical architecture or simply the output for the proposed one in Figure 1), and a convolutional layer output $A\in R^{K\times U\times V}$ (where we extract the matrix $A_{k}\in R^{U\times V}$ relative to the channel k∈{1,⋯,K} - U,V are the output matrices dimensions for any of the *k* channels), then the gradient $G_{y_{n},A_{k}}\in R^{U\times V}$ of $y_{n}$ with respect to $A_{k}$ is defined as

(7) $$\begin{array}{c} G_{y_{n},A_{k}}=\frac{\partial y_{n}}{\partial A_{k}}. \end{array}$$

The importance weight of the channel *k* with respect to the class *y* is naturally obtained as the average $\alpha_{y,k}$ across the convolutional layer matrix entries

(8) $$\begin{array}{c} \alpha_{y,A_{k}}=\frac{1}{UV}\sum_{i=1}^{U}\sum_{j=1}^{V}G_{y_{n},A_{k},i,j}. \end{array}$$

Finally, the Grad-CAM map with respect to a target *y* is defined as $L_{y}$, namely the weighted sum across all the dimensions *k*, is

(9) $$\begin{array}{c} L_{y}=\mathrm{ReLU}\left(\sum_{k=1}^{K}\alpha_{y,A_{k}}A_{k}\right), \end{array}$$

where the ReLU function [127] simply suppresses all the negative values highlighting the interest for the features that have only a positive influence towards a certain target.

Since the first part of the architecture is unencrypted (see Figure 1b), the network can be inspected easily by either the user (using its own private data) or the model designer (using a set of data not constrained by privacy issues) both when the network parameters are encrypted or unencrypted.

In our work, we extract the Grad-CAM attention maps relative to the last convolutional layer of VGGNet. Usually, earlier convolutions extract low-level features (e.g., edges or corners), while deeper convolutional layer are able to describe more abstract features, such as geometrical shapes or complex connected regions [79], which are extremely significative for tasks such as facial or image recognition. Note that Grad-CAM allows inspecting the network perception even if some of the deep layers are kept fixed and just the last layers are modified or fine-tuned (like in our case, see Figure 1b). In fact, the perception is propagated from the output to the inner convolutional layers, which allows to track back changes on the last weights [79].

Although gradient-based methods might not be the optimal solution for visual explanation (e.g., saturation, zero-gradient image regions, and false confidence in the output score phenomena [156]), the computational cost of Grad-CAMs is negligible when compared to other methods that require multiple network forward-passes per image [156,157]. Moreover, Grad-CAM is considered the reference method in several recent works [157,158,159,160,161].

The second implemented approach to globally explain the deep network behavior consists in observing whether the network maps the input data into a feature space able to both preserve performances and mask the membership in a protected population. Fixing an internal network layer, this task can be performed by reducing the dimensionality of the layer’s original space to a lower-dimensional (possibly two) and more interpretable one. In our work we rely on the t-SNE algorithm [80,81] for effectively carrying out this dimensionality reduction. As an unsupervised approach, it allows one to evaluate the statistical distribution of the extracted features hiding task-related information, which may produce undesired distortions.

t-SNE firstly calculates the similarity between points both in the high-dimensional space and in the corresponding low-dimensional one. The similarity is calculated as the conditional probability that a point $P_{1}$ would choose point $P_{2}$ as its neighbor following a Gaussian distribution centered at $P_{1}$. Then, it tries to minimize the difference between these conditional probabilities in the higher-dimensional and lower-dimensional spaces by minimizing the sum of Kullback–Leibler divergence of overall data points using a gradient descent method.

In this work, we applied directly t-SNE on the 16-dimensional embedding (since it is the only one which varies with the training phase) for the proposed architecture (see Figure 1b).

For what concerns the classical architecture (see Figure 1a), instead, the 25,088-dimensional embedding is too big to be fed directly to the t-SNE algorithms. For this reason, we will adopt a two-step approach for effectively reducing its dimensionality. The first step of this feature reduction is supervised (by means of $L_{1}$-regularized Logistic Regression [162]), while the second one is un-supervised (by means of the t-SNE). The first step allows us to remove the features with zero contributions to the specific task under examination. The second step allows us instead to evaluate the statistical distribution of the remaining features hiding task-related information which may produce unwanted distortions. As usually happens in deep networks, the representation vector has a large number of elements (to allow its use in multiple tasks) but only a subset of them is needed to solve a specific task. Exploiting a $L_{1}$-regularized Logistic Regression allows to discard the features with no contribution to the task solution (by means of the $L_{1}$-regularization [163]) reducing the dimension of the space to just the informative features for the considered task. Since t-SNE is a computationally demanding algorithm, usually a PCA-based [164] pre-dimensionaly reduction step is adopted.

## 5. Experimental Results

In this section we present the results of applying the methodology presented in Section 4 on the FairFace real-world dataset [65]. In particular, in Section 5.1 describes the FairFace dataset. Then, Section 5.2 describes the architectural configurations tested in the study (i.e., with and without HE and/or fairness constraints). Section 5.3 reports the performance of this architecture in terms of accuracy and fairness, while Section 5.4 focuses on their computational requirements. Finally Section 5.5 and Section 5.6 focus on local and global explainability, respectively, to give more insights into what the different architecture actually learned from the data and what the effects of introducing privacy and fairness requirements are. All the codes for producing the results are made freely available to the community (https://github.com/lucaoneto/ENTROPY_2021 (accessed on 11 August 2021)).

### 5.1. The Dataset

The FairFace dataset [65] is a collection of ≈100 thousand facial images extracted from the YFCC-100M Flickr dataset [165]. Automated models trained on FairFace can exploit age group (age ranges of [0–2], [3–9], [10–19], [20–29], [30–39], [40–49], [50–59], [60–69], and [70+]), gender (which, for this dataset, refers to the perceived binary gender (Male and Female) of an individual), and ethnicity (Western White, Middle Eastern White, East Asian, Southeast Asian, Black, Indian, and Latinx.) as sensitive information. Our task consists in predicting whether a face belongs to a person with more (1) or less (0) than 30 years old measuring the discrimination between individuals with different gender. Table 2 reports some statistics about the FairFace dataset with respect to the selected sensitive attribute.

For this work, the training and test sets are composed, respectively, of 86.7 thousand and 10.9 thousand images (same split of the original FairFace dataset [65]).

### 5.2. Tested Configurations

In this section, we summarize all the architecture that we tested in the experiments:

- The classical VGGNet-16-based face recognition architecture (see Figure 1a) under the following settings:
  – The architecture was trained with a random selection of 20,000 training and 10,000 test images from the training and test sets, respectively. We train every model for a total of 10 epochs using the ADADELTA [166] gradient descent method with mini batches of 150 images. The layers before the last convolution one (excluded)were not fine-tuned and would benefit from the parameters pre-trained on the VGG-Face dataset (see Section 4.2);
  – We investigated the case when the fairness constraint (see Section 4.3) is or is not imposed in the last convolutional layer;
  – We also derived the attention maps and the dimensionality reduction with respect to the last convolutional layer.
- The proposed VGGNet-16-based facial recognition architecture (see Figure 1b) under the following setting:
  – The architecture was trained with a random selection of 1000 training (because of the limitation imposed by HE; see Section 4.1) and 10,000 test images from the training and test sets, respectively. We trained every model for a total of 10 epochs using gradient descent [66]. Before the actual training could take place, the embeddings needed to be reduced to a much smaller representation vector due to the computational limitation imposed by HE (see Section 4.1). To perform this task, we trained the architecture depicted in Figure 1b without applying HE. We chose a 32-dimensional representation since it represents a good tradeoff between information compression (due to the HE limitations) and utility (the accuracy of the whole network remains unaltered). This preliminary phase observes the same settings imposed for training the classical architecture. Once the network parameters are trained for extracting the 32-dimensional representation vector, we reset the weights of the last two dense layers following again the original Gaussian distribution N(0,0.01). This simulates the case when a new network is trained from scratch by applying privacy guarantees through HE. The layers before the last convolution one (excluded) were fine-tuned and could benefit from the parameters pre-trained on the VGG-Face dataset (see Section 4.2).
  – We investigate the case when HE was or was not exploited (see Section 4.1) in the last three layers of the network (see Figure 1b).
  – We investigated the case when the fairness constraint (see Section 4.3) was or was not imposed in the last hidden layer;
  – We derived the attention map with respect to the last convolutional layer. We applied, instead, the dimensionality reduction to the last hidden layer.

### 5.3. Accuracy vs. Difference of Demographic Parity

In this section, we evaluate the different architectures in terms of accuracy ACC and fairness DDP, on the test set.

**Figure 2:**
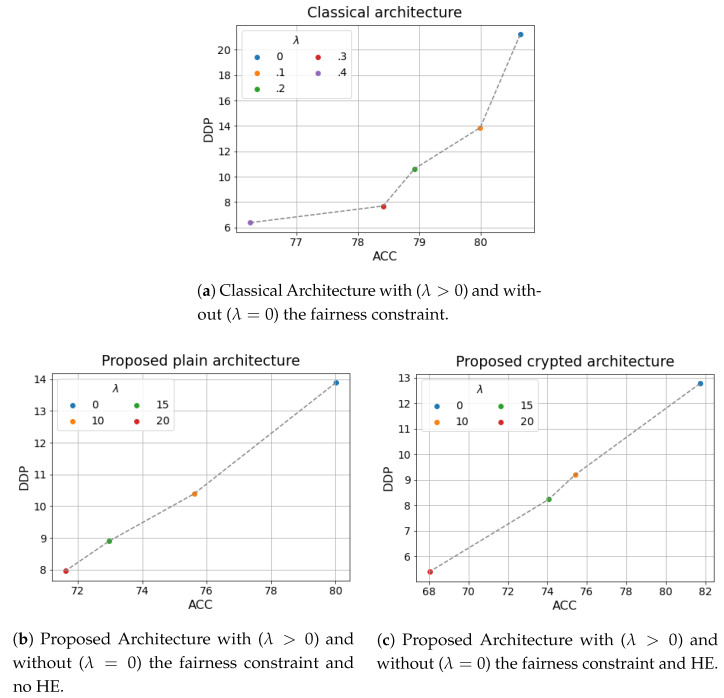
Comparison between the different architectures in terms of accuracy ACC and fairness DDP.

In particular, Figure 2 reports the ACC against the DDP for the different architectures (see Section 5.2) when different values of λ are exploited.

Two tendencies can be observed in Figure 2. The first one refers to the tension between accuracy and fairness: the more fair we want the model to be (the higher value for the regularization parameter λ), the less accurate the model will be on the available data (i.e., data are biased and then, trying to remove these bias, not fully trustable). The second one refers to the tension between accuracy and privacy: enforcing privacy with HE actually reduces our ability to use large amounts of data, computation, and architectural choices and hence reduces our ability to learn accurate models. Nevertheless, the results of the proposed architecture gives similar results as expected from the theory (see Section 4.1) whether HE is present or not, while the small differences are obviously due to the noise introduced by HE in the computation.

Figure 2 clearly shows the effectiveness of the proposed approaches in learning private and fair models.

### 5.4. Computational Requirements

In this section, we would like to report and underline the computational requirements for training the different architectures under examination (see Section 5.2).

In particular, Table 3 reports the training time and memory requirements averaged over different runs for the different architectures described in Section 5.2. Experiments were run on a machine equipped with Windows Server 2019, 4 Intel Xeon CPU E5-4620, 256GB DDR3 of RAM, 1 TB SSD disk, Python 3.7, scikit-learn 0.24.2, PyTorch 1.8, and TenSeal 0.3.4.

From Table 3, it is possible to note the explosion, in terms of computational requirements, when the HE is employed. This is expected from the theory (see Section 4.1), and this is the reason behind the architectural choices (the reduction of the embedding dimension from 25,088 to 32) and the limitation in the size of the training set from 20,000 to 1000). Nevertheless, the results of Section 5.3 have shown how these limitations actually do not compromise the ability to learn fair and accurate models.

### 5.5. Attention Maps

In this section, we aim at assessing a possible discriminatory attention behavior carried out by the different architectures (see Section 5.2) tested in Section 5.3 and Section 5.4.

In fact, following the method presented in Section 4.4, we wish to observe whether the application of the fairness constraint produces less discriminatory attention mechanisms, namely more similar attention maps between different subgroups. In order to standardize the image face regions, we exploited a set of 50,000 images of frontal faces extracted from the Diversity in Faces dataset [167], where, again, gender was exploited as the sensitive feature.

Firstly, we took the average attention maps of both males and females, and we computed the difference between these two average attention maps through the Frobenius distance [168]. More formally, for each image in the dataset, let us compute the grad-CAM attention map $L_{Y}$ (see Section 4.4). Then, let us define $M_{s}\in R^{U\times V}$, with s∈{males,females}, as the dataset averaged $L_{Y}$ for each subgroup in the population. Finally, the Frobenius distance of $M_{\mathrm{males}}$ and $M_{\mathrm{females}}$ is computed as

$$\begin{array}{c} \mathrm{FRO}\left(M_{\mathrm{males}},M_{\mathrm{females}}\right)=\sqrt{\sum_{i=1}^{U}\sum_{j=1}^{V}{\left(M_{\mathrm{males},i,j}-M_{\mathrm{females},i,j}\right)}^{2}}. \end{array}$$

**Figure 3:**
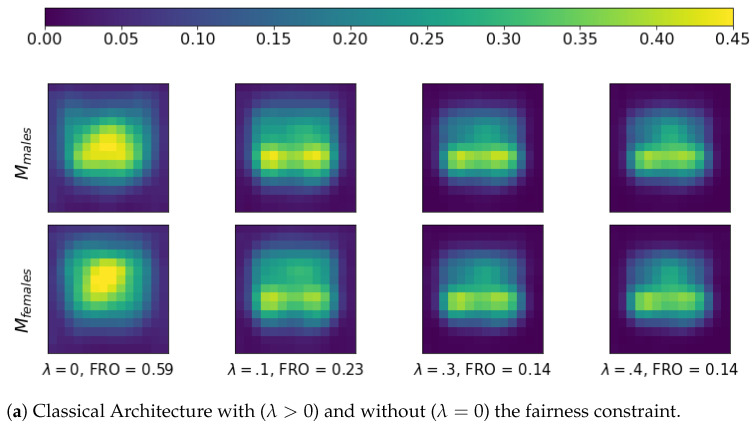

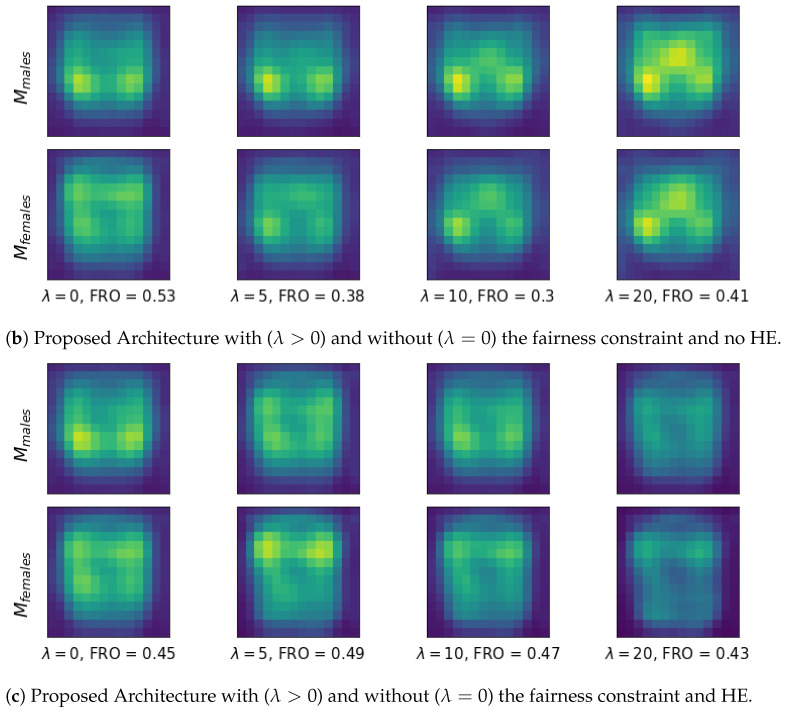
Comparison between the different architectures using the average attention map extracted with Grad-CAM.

Figure 3 reports $M_{\mathrm{males}}$, $M_{\mathrm{females}}$, and $\mathrm{FRO}(M_{\mathrm{males}},M_{\mathrm{females}})$ for the different architectures with (λ>0) and without (λ=0) the fairness constraint. Figure 3 clearly shows the positive effect of the fairness regularizes in reducing the networks’ discriminatory attention mechanism, which is quite evident if compared to the no-regularized case.

**Figure 4:**
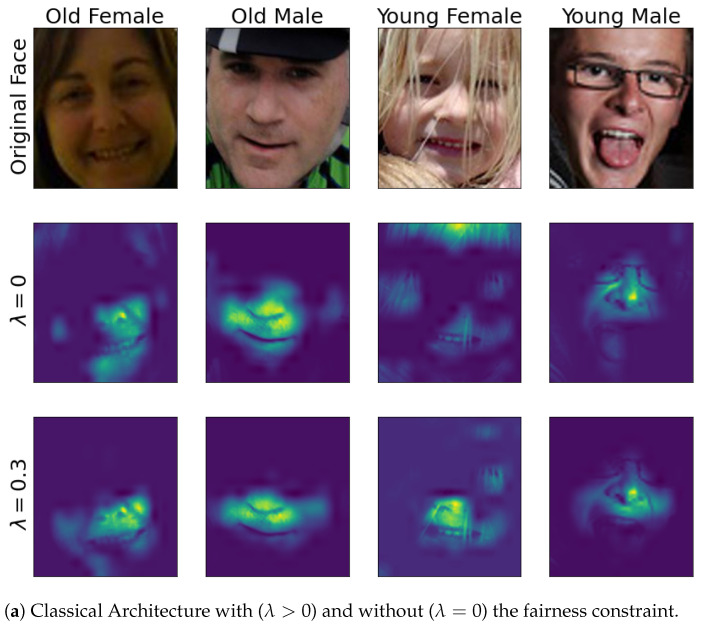

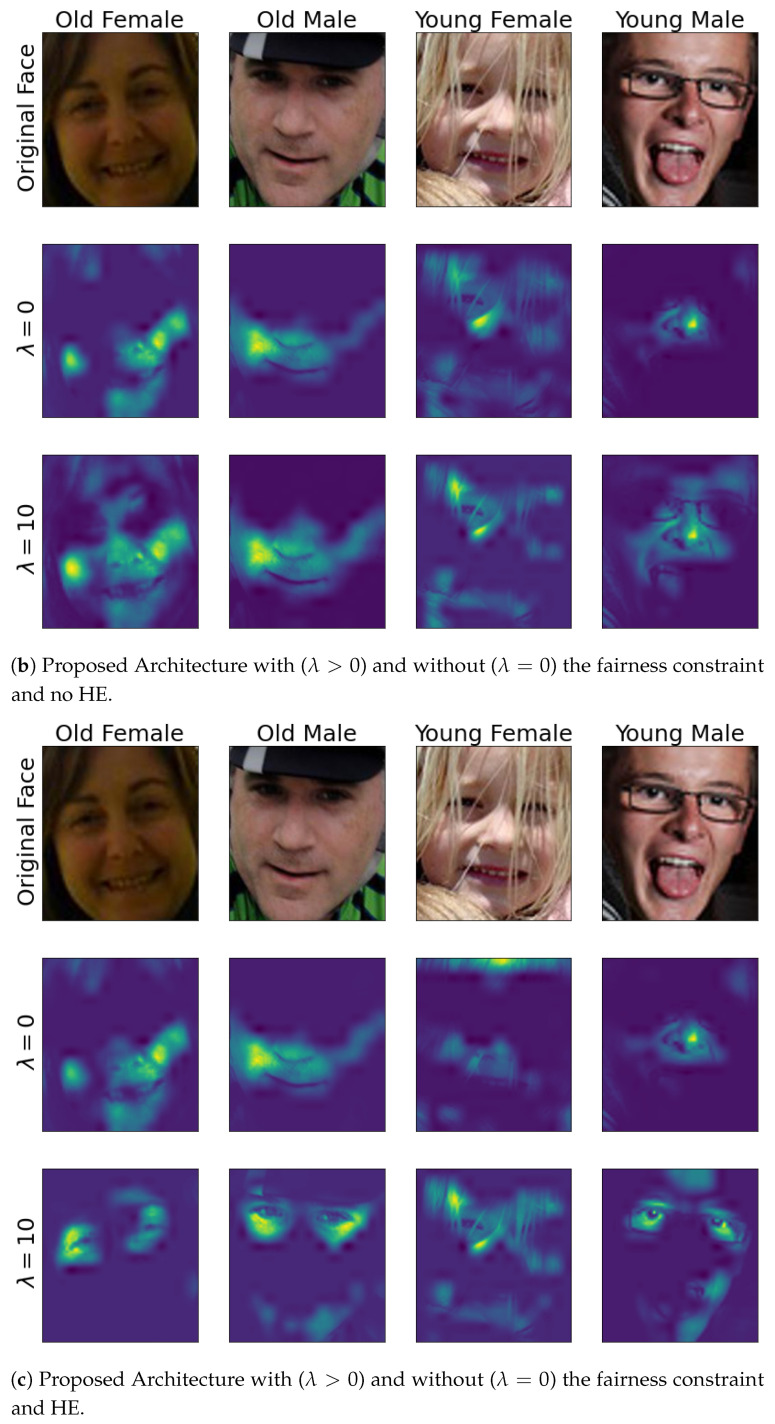
Comparison between the different architectures using the attention map of sample images extracted with Grad-CAM.

For sake of completeness, we also report in Figure 4 the attention map for the different architectures with (λ>0) and without (λ=0) the fairness constraint for a young male, a young female, an old male, and an old female. Due to space limitations, we report just the results with a λ that showed the best accuracy/fairness in the results of Section 5.3.

Figure 4 clearly shows how the fairness regularizer is able to restrict the networks’ receptive field to class-specific face regions.

### 5.6. Dimensionality Reduction

In this last set of experiments, we analyzed the distribution of the representation vectors (see Section 5.2 and Figure 1) by means of dimensionality reduction using the pipeline presented in Section 4.4.

For what concerns the classical architecture (Section 4.4), we cross-validated the $L_{1}$-regularization strength in the $L_{1}$-regularized logistic regression obtaining a top accuracy above 80% for the binary classification task. This step discarded most of the representation vector features extracted by the VGGNet-16, keeping just ≈450 features (i.e., those with weights different from 0). Then, the PCA further reduced the dimensionality of the space from ≈450 to 50 features. Finally, the t-SNE has been exploited to map this 50-dimensional space into a 2-dimensional space.

For what concerns the proposed architectures (Section 4.4), instead, the t-SNE was exploited to directly map the hidden 16-dimensional embedding into a 2-dimensional space.

**Figure 5:**
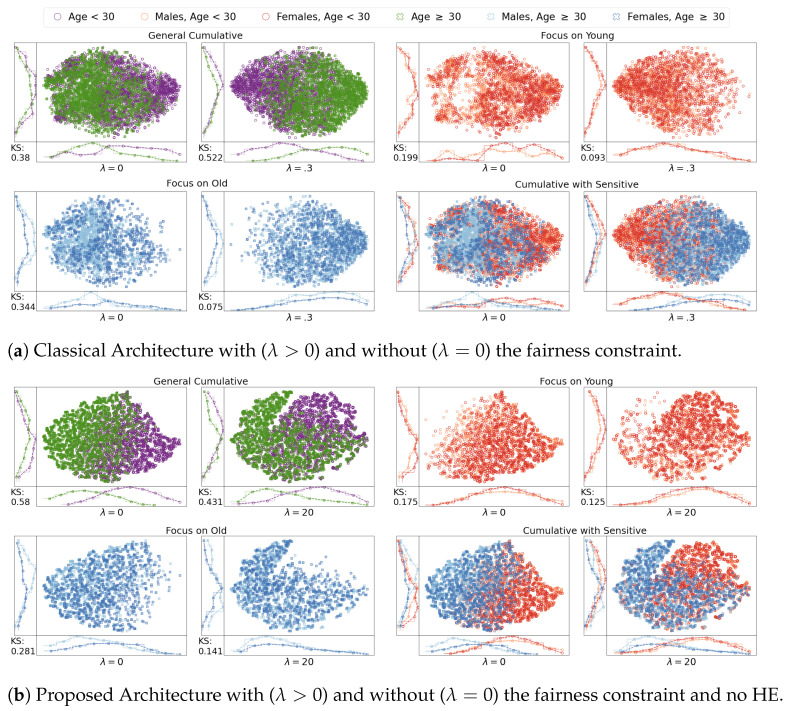

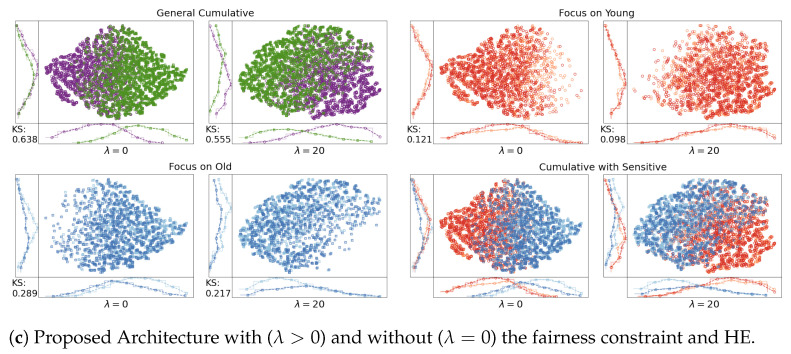
Comparison between the different architecture using the t-SNE dimensionality reduction algorithm on the learned embedding.

Figure 5 displays the results for the different architectures, with (λ>0) and without (λ=0) the fairness constraint, on a random selection of 3000 samples from the test set to allow a friendly visualization. Due to space limitations, we report just the results with a λ, which showed the best accuracy/fairness in the results of Section 5.3. For the sake of completeness, Figure 5, also reports the two-dimensional Kolmogorov–Smirnov (KS) distance [169,170] between the distributions of Males and Females in this low-dimensional space. Figure 5 clearly shows how the application of the fairness regularizer reduces the amount of discrimination: Males and Females are distributed similarly in the space after the application of the fairness regularizer, while before they either were clustered in different sub-spaces (Figure 5a) or presented a higher KS distance (Figure 5a–c). This means that the regularizer reduced the ability to identify Males and Females simple based on their position in the space defined by the representation vector learned by the architecture.

## 6. Conclusions

The use of artificial intelligence coupled with the ability to learn from historical data is becoming ubiquitous. For this reason, the social and ethical implications of the widespread use of tools empowered with such intelligence cannot be ignored any longer. The increasing concerns regarding these issues are not only increased by the population or by the institutions, but also by researchers who have shown potential discriminatory behavior, by threats to privacy and to the right of explanation, and by risks of attacks in current artificial intelligence systems. Institutions such as the European Union have created a high-level expert group on this subject drawing guidelines for more trustworthy intelligent systems (https://digital-strategy.ec.europa.eu/en/policies/expert-group-ai (accessed on 11 August 2021)).

For this purpose, in this work, we work toward the development of systems able to ensure trustworthiness by delivering privacy, fairness, and explainability by-design. In particular, we have shown that it is possible to simultaneously learn from data while preserving the privacy of the individuals thanks to the use of Homomorphic Encryption, ensure fairness by learning a fair representation from the data, and ensure explainable decisions with local and global explanations without compromising the accuracy of the final models. We then tested the practicality of our approach on a widespread and controversial application, namely the face recognition, using the recent FairFace dataset to prove the validity of our approach.

To the best knowledge of the authors this is one of the first results in this framework with actual practical results. Nevertheless, this work is just a step forward toward the design of fully trustworthy intelligent systems. For example, in the future, more applications could be explored. Moreover, we need to address the requirement of robustness, which demands making the approach more robust to the presence of adversarial attacks (i.e., adversarial samples or poisoning methods). For this aspect, our framework is already designed to encapsulate robustness requirements since adversarial defense mechanisms are mostly based on gradient-based methods, which marry well with our framework. Finally, while a strong theoretical framework has been developed for the different methods employed in this work, a theoretical framework able to simultaneously offer statistical guarantees of privacy, fairness, and explainability still needs to be designed. 

## Figures and Tables

**Table 1:** Notations.

Symbol	Description
I	General input space of RGB images
*h*	RGB images height
*w*	RGB images width
S	Binary sensitive attribute space
*s*	s${}^{\mathrm{th}}$ sensitive group
Y	Binary label space
D	Full dataset
$D_{s}$	Samples from D in the s${}^{\mathrm{th}}$ sensitive group
Z	Model input space that may (or not) contain the sensitive attribute
*Z*	Model input
h	General end-to-end model
$h^{*}$	Learned end-to-end model
r	Sub-model that learn the data representation (embedding layers)
m	Sub-model that learn the task from the representation (task specific layers)
P	General utility measure
ACC	Accuracy Measure
ϕ	Homomophic map
A, B	Sets with same algebraic structure
*p*	Degree of the encoded cyclotomic polynomial
F	General fairness measure
λ	Fairness regularization hyper-parameter (Tikhonov formulation)
η	Fairness regularization hyper-parameter (Ivanov formulation)
DP	Demographic Parity
DDP	Difference of Demographic Parity
AVG	First order (convex and differentiable) approximation of the DDP
*y*	Classification prediction target
$y_{n}$	Non-normalised classification model prediction for *y*
*A*	Convolutional layer output
$A_{k}$	Matrix relative to channel *k* in layer output *A*
$G_{y_{n},A_{k}}$	Gradients matrix of $y_{n}$ w.r.t. $A_{k}$
$\alpha_{y,A_{k}}$	Importance weight of $A_{k}$ w.r.t. the target *y*
$L_{y}$	Grad-CAM map w.r.t. the target *y*
$M_{s}$	Dataset average Grad-CAM for the s${}^{\mathrm{th}}$ sensitive group
FRO	Frobenius distance
KS	Kolmogorov–Smirnov distance

**Table 2:** Fairface: label distribution (gender is the sensitive features).

	Age ≥30	Age <30	*Sensitive* *Marginals*
**Females**	18.60% 18,174	28.40% 27,746	47.00% 45,920
**Males**	27.21% 26,587	25.79% 25,191	53.00% 51,778
*Class* *Marginals*	45.82% 44,761	54.18% 52,937	97,698

**Table 3:** Training time and memory requirements averaged over different rounds for the different architectures.

Architecture	Fairness Constraint	Homomorphic Encryption	Number of Training Samples	Training Time (sec/epoch)	Memory Requirements (GB)
Classical			20,000	6200	≈10
(Figure 1a, full)	x		(batches of 150)	6100	≈10
Proposed			1000	<1	≈1
(Figure 1b,	x			<1	≈1
training last two		x		2100	≈30
dense layers)	x	x		3000	≈30

## Data Availability

In this work we exploited the FairFace dataset [65]. The code is available at the following link https://github.com/lucaoneto/ENTROPY_2021 (accessed on 11 August 2021).
